# Water Supply

**Published:** 1911-04-08

**Authors:** 


### SPECIAL INSTITUTIONAL ARTICLE.

### WATER SUPPLY.

### III.—STORAGE.

Having described the methods of creating supply and the sources thereof, the next consideration is the means of storage. Well water is usually hard, and though useful for drinking purposes, is not so desirable for laundry work, but as rainwater is eminently soft, it would be advantageous to store this for laundry and other administrative purposes. The first rainwater from a roof after a long period of dry weather is usually of an impure nature on account of soot, bird excrement, etc., and in order to prevent this portion from entering the storage tanks mechanical devices known as "separators" are used; these consist of vessels into which the rainfall is directed before passing into the storage tanks, and which are so made as to collect the first portion of the flow, and when full to cant over, emptying their contents to waste and at the same time placing the flow of water in direct communication with the storage tanks. When the flow has ceased they return automatically to the original position. Rain water may be stored either above or under ground. The former method has the advantage of enabling water to be drawn by means of a tap, whereas the latter, while keeping the water cool, will in most cases necessitate pumping and, unless great care is taken, be more liable to contamination. The water should in either case be run over a copper grid to keep back dead leaves and other vegetable or organic matter before entering the storage tank, and should also pass through an efficient filter. The filtering material may be sand, polarite or other gritty substance, and the tank should be well ventilated, having good access for its easy and thorough cleansing.

When the available roof surface is not adequate, a small area of ground may be drained either by means of agricultural drains or by the formation of asphalte yards, and the water taken by means of piping to the filtering bed and thence to the storage tanks.

The allowance of water for all purposes per person per diem may be reckoned at from twenty to thirty gallons, and as the average rainfall for great Britain stands at 33 inches per annum,* it is an easy matter to calculate the collecting area required for supplying any given number of persons with the desired proportion of rain water. A rough and ready formula for ascertaining the amount of gallons obtainable per annum from a determined area is to multiply the square feet in that area by half the number of inches in the annual average rainfall of the district. For example, in a county where the annual average rainflal is 26 inches a collecting area of 5 by 2 feet will yield annually 130 gallons. In measuring the area the dimensions must be strictly horizontal.

It is generally admitted that the amount of storage should equal the amount of consumption for twenty-four hours, therefore in an establishment of say fifty persons at say twenty-five gallons per diem, we shall require 1,250 gallons. This should be stored in the roof or in tanks on the roof, so as to get as high a pressure as possible. When the tanks stand externally on the roof they should be covered by non-conductive materials, though of easy access for cleaning purposes. A tank to take, say, 1,500 gallons, would be about 10 feet square, by 3 feet high. It is desirable to have shallow tanks, unless a separate water-tower is specially built, in order to distribute the weight as much as possible. The best type of tank for such a size is that built up of steel plates with broad flanges inside, bedded in composition of a particular kind, and with stiffening cross-ties. The steel plates of which such tanks are built are about four superficial feet in area. The tank should rest on rolled steel joists in order to distribute the weight as much as possible, and should have a tray of lead or asphalte underneath as a precaution against leakage.

The foregoing remarks have applied to the question of capacity in overhead tank storage to which the water is pumped from some lower reservoir. The storage capacity of the reservoir itself has to be calculated on a more problematic basis, involving consideration of the probable periods between rainstorms and the probable amount of rain water liable to be secured from a single downpour.

If the annual rainfall were equally divided through the months so that we might expect a minimum of two inches in each month, it would be easy to dispense with large storage. But as the records show that we may sometimes expect a fall of 4 inches in less than three weeks, preceded and followed by several weeks of comparative drought, it is clear that it is unwise to provide for less storage capacity than one-sixth of the annual yield. Otherwise there may be both waste and want.

The important question of the softening of waters which have been hardened by contact with different soils is a subject which it is proposed to treat in a separate and subsequent article.

* Thirty-three inches is the average for the British Isles, but as this average is brought up by the exceptional rainfall in certain particclar districts, it is wiser to ascertain, if possible, the actual figure for the district concerned; near London the average fal is about 25.

